# Combinatorial immunotherapy with gemcitabine and ex vivo-expanded NK cells induces anti-tumor effects in pancreatic cancer

**DOI:** 10.1038/s41598-023-34827-z

**Published:** 2023-05-11

**Authors:** Eun-Kyoung Koh, Hong-Rae Lee, Woo-Chang Son, Ga-Young Park, Juhee Kim, Jae-Ho Bae, You-Soo Park

**Affiliations:** 1 Department of Research Center, Dongnam Institute of Radiological and Medical Sciences, Busan, 46033 South Korea; 2 Department of Biochemistry, Pusan National University School of Medicine, Yangsan, 50612 South Korea

**Keywords:** Cancer, Immunology

## Abstract

Pancreatic cancer is difficult to diagnose at the initial stage and is often discovered after metastasis to nearby organs. Gemcitabine is currently used as a standard treatment for pancreatic cancer. However, since chemotherapy for pancreatic cancer has not yet reached satisfactory therapeutic results, adjuvant chemotherapy methods are attempted. It can be expected that combining immune cell therapy with existing anticancer drug combination treatment will prevent cancer recurrence and increase survival rates. We isolated natural killer (NK) cells and co-cultured them with strongly activated autologous peripheral blood mononuclear cells (PBMCs) as feeder cells, activated using CD3 antibody, IFN-r, IL-2, and γ-radiation. NK cells expanded in this method showed greater cytotoxicity than resting NK cells, when co-cultured with pancreatic cancer cell lines. Tumor growth was effectively inhibited in a pancreatic cancer mouse xenograft model. Therapeutic efficacy was increased by using gemcitabine and erlotinib in combination. These findings suggest that NK cells cultured by the method proposed here have excellent anti-tumor activity. We demonstrate that activated NK cells can efficiently inhibit pancreatic tumors when used in combination with gemcitabine-based therapy.

## Introduction

Pancreatic ductal adenocarcinoma (PDAC), the most common pancreatic malignancy, accounts for more than 90% of all pancreatic cancers^1^. PDAC is the seventh leading cause of cancer-related death in both men and women and has a poor prognosis^2^. The 5-year survival rate from PDAC varies by region and country, but does not exceed 10%^3^. Due to the special anatomical location of the pancreas, PDAC is generally diagnosed late stage of cancer when there are already obvious clinical symptoms^4^. Surgical resection is potentially the only treatment option in patients with early PDAC, but many patients (> 80%) already have metastatic or recurrent conditions at initial diagnosis, when surgery is already inappropriate^5,6^.

Gemcitabine-based chemotherapy has been introduced for unresectable metastatic PDAC^7^. Several studies have reported on combination therapies that can increase the objective response rate and overall survival (OS) of patients. Various clinical studies have indicated that gemcitabine-based combinations are more effective than gemcitabine alone, and erlotinib was the first drug to significantly improve survival. Erlotinib was approved by the US Food and Drug Administration in 2005 for treating local progressive or metastatic PDAC^8^. Combining gemcitabine and erlotinib prolonged patient OS by only 0.33 months relative to gemcitabine monotherapy^8^. Erlotinib is an epidermal growth factor receptor (EGFR) tyrosine kinase inhibitor; there is controversy over its low efficacy, given that abnormalities in the EGFR pathway in PDAC are uncommon, and that most *K-ras* mutations occur in a sub-pathway^9,10^. A 2011 clinical study reported that combining gemcitabine and FOLFIRINOX therapy prolonged OS by 4.3 months relative to gemcitabine alone^11^. A 2013 clinical study reported that combining gemcitabine and nab-paclitaxel prolonged OS by 18 months relative to gemcitabine alone^12^. However, since these combination therapies were limited to patients with advanced pancreatic cancer in relatively good health^13,14^, there is increasing interest in immune cell therapy that can overcome the toxicity or side effects of anticancer drugs^15^.

Immunotherapies are being evaluated in various ways as a new clinical treatment strategy for PDAC^15^. Checkpoint inhibitors, tumor vaccines, and the adoption of cell therapy have the potential to improve existing treatments for PDAC^16–19^. In particular, reduced natural killer (NK) cell activity has been observed in patients with PDAC, and reactivated NK cells or therapy using highly active NK cells may be potential treatments for pancreatic cancer^20^. NK cells are cytotoxic lymphocytes that play important roles in the innate immune response by eliminating virus-infected, abnormal, and cancer cells without recognizing specific antigens in advance^21,22^. NK cells are highly sensitive against tumor cells with reduced or no expression of major histocompatibility complex (MHC) class I molecules. In contrast, NK cells have low sensitivity toward tumor cells that express high levels of MHC class I molecules^22,23^. This is because MHC class I in tumor cells binds to the inhibitory receptors of NK cells^24^. Natural killer group 2 member D (NKG2D) is a typical activation receptor, a type of receptor that recognizes ligands expressed when tumor cells are in an abnormal state and activates NK cells^25^. NK cell function is regulated by a balance between the activation and inhibition of receptors that bind to various ligands on the target-cell surface. Therefore, a strong activation signal is required to overcome these limitations.

It has been reported that chemotherapeutic agents induce the expression of various NK cell-activating ligands on tumor cells; these ligands include NKG2D ligands, Fas (apoptosis-stimulating Fragment), poliovirus receptor (CD155), Nectin-2 (CD112), B7-H6 (B7 family member), and TRAIL (tumor necrosis factor-α-related apoptosis-inducing ligand)-R1/R2. In particular, NKG2D ligands are important factors in increasing the NK response to tumor cells. The expression of NKG2D ligands induced by gemcitabine and erlotinib varies among tumor cells^26–33^.

In a preclinical model of PDAC, a study comparing immune cell proportion in the resection margin after adjuvant gemcitabine treatment showed a significant increase in the number of NK cells but not CD8^+^ T cells. In addition, NK cell depletion increased the risk of local recurrence and prevented extended survival^34^. Therefore, gemcitabine treatment for PDAC could be a strategy to activate NK cell-mediated anti-tumor responses. Although studies on gemcitabine and NK cells have been reported, combining gemcitabine and erlotinib with NK cells has not been well-studied. Therefore, we applied a combination therapy comprising highly cytotoxic ex vivo-expanded NK cells, gemcitabine, and erlotinib to treat pancreatic cancer more effectively than the standard regimen. In a non-obese diabetic/severe combined immunodeficiency (NOD/SCID) mouse model of human pancreatic cancer, we demonstrated that this multi-mode approach effectively inhibited the growth of pancreatic cancer cells, resulting in improved anti-tumor effects.

## Materials and methods

### Ethical approval

Experiments using all human study samples (human blood) were approved by the ethics committee of Dongnam Institute of Radiological and Medical Sciences (DIRAMS), and all procedures were performed according to the relevant guidelines and regulations approved by the Institutional Review Board (IRB) at DIRAMS. All participants gave written informed consent in accordance with the Declaration of Helsinki (IRB approval No.: D-1508-002-001, D-2022-032-002).

Specific pathogen-free (SPF) mice were maintained in a laminar airflow cabinet. All necessary precautions were taken to minimize the number of animals used and to alleviate pain. Animal procedures were performed according to the protocol approved by the DIRAMS Institutional Animal Care and Use Committee (IACUC; Approval No.: DIRAMS AEC-2019-006). All methods were carried out in accordance with the relevant guidelines and regulations and the ARRIVE guidelines (https://arriveguidelines.org/).

### Cell culture

K562 (CCL-243), PANC-1 (CRL-1469), and MIA PaCa-2 (CRL-1420) cells were purchased from the American Type Culture Collection (ATCC, Manassas, VA, USA). The K562 cell line was maintained in RPMI 1640 medium and the PANC-1 and MIA PaCa-2 cell lines were maintained in DMEM medium supplemented with 10% (v/v) fetal bovine serum (FBS, Gibco, Thermo Fisher Scientific, Waltham, MA, USA), Gibco 1× Anti-Anti solution (Thermo Fisher Scientific), and were maintained at 37 °C in a humidified atmosphere containing 5% of CO_2_.

### WST-based cell viability assay

The WST-based cell viability assay was conducted using an EZ-CYTOX kit (DoGenBio, Seoul, South Korea). Briefly, 100 μL of PANC-1 cells were plated in 96-well plates at 8 × 10^3^ cells/well, and 100 μL of MIA PaCa-2 cells were plated at 5 × 10^3^ cells/well. After overnight adherence, the cells were treated with the indicated concentrations of chemotherapeutic agents and incubated for 24, 48, or 72 h. Thereafter, 10 μL of EZ-CYTOX solution was added into each well, and the plate was incubated for another 3 h. The absorbance of the solution was assessed using a spectrometer (VersaMax plate reader, Molecular Devices, San Jose, CA, USA) at 450 nm.

### NK cell isolation and expansion

PBMCs was isolated from whole peripheral blood via density gradient centrifugation using Histopaque-1077 (Sigma-Aldrich, MO, USA). To obtain high-purity NK cells, non-NK cells were depleted using an EasySep Direct Human NK Cell Isolation Kit (STEMCELL Technologies, Vancouver, BC, Canada) according to the manufacturer’s instructions. NK cell purity was evaluated via flow cytometry using anti-human CD3 (BD Biosciences, San Jose, CA, USA) and anti-human CD56 monoclonal antibodies (BD Biosciences). Highly purified NK cells were seeded at a density of 1 × 10^5^ cells/mL in 24-well culture plates coated with 5 µg/mL anti-human CD16 mAb (clone B73.1; eBioscience, San Diego, CA, USA).

Autologous PBMCs were irradiated with 25 Gy γ-radiation after > 30 min stimulation via 0.5 μg/mL anti-Human CD3 antibody (clone OKT3; eBioscience), 1000 U/mL of recombinant human IFN-γ GMP (R&D Systems, Minneapolis, MN, USA) and 1000 U/mL of recombinant human IL-2 (PROLEUKIN, Boehringer Ingelheim, Vienna, Austria). Stimulated autologous PBMCs were seeded at a density of 2 × 10^6^ cells/mL and co-cultured with purified NK cells. The culture medium was Lymphocyte Growth Medium-3 (LGM-3, Lonza, Walkersville, MD, USA) containing 500 U/mL of recombinant human IL-2 (PROLEUKIN) and 5% (v/v) human serum (Biowest, Riverside, MO, USA). NK cell expansion was performed under Good Manufacturing Practice (GMP) conditions for 21 days.

### Flow cytometry

The phenotype of NK cells and cancer-cell NKG2D ligand were assessed via flow cytometry on an FC 500 flow cytometer (Beckman Coulter, Fullerton, CA, USA) with the following monoclonal antibodies against human proteins: FITC Mouse IgG1, κ (Clone MOPC-21), FITC Mouse Anti-Human CD3 (clone UCHT1), FITC Mouse Anti-Human CD226 (DNAM-1; Clone DX11), FITC Mouse Anti-Human NKB1 (Clone DX9), FITC Mouse Anti-Human CD1598b (Clone CH-L), FITC Mouse IgG2a, κ (Clone G155-178), FITC Mouse Anti-Human CD244 (2B4; Clone 2–69), FITC Mouse Anti-Human CD1598a (Clone HP-3E4), PE Mouse IgG1 (Clone MOPC-21), PE Mouse Anti-Human CD3 (Clone UCHT1), PE Mouse Anti-Human CD16 (Clone 3G8), PE Mouse Anti-Human CD335 (NKp46; Clone 9E2), PE Mouse Anti-Human HLA-ABC (Clone G46-2.6), PE Mouse IgG1, κ (Clone MOPC-21), PE Mouse Anti-Human CD336 (NKp44; Clone p44-8), PE Mouse Anti-Human CD253 (TRAIL; Clone RIK-2), PE Mouse Anti-Human CD337 (NKp30; Clone p30-15), PE Goat Anti-Mouse Ig (Multiple Adsorption; Clone Polyclonal), PE-Cy5 Mouse IgG1, κ (Clone MOPC-21), and PE-Cy5 Mouse Anti-Human CD56 (Clone G44-26), purchased from BD Biosciences. PE Mouse Anti-Human CD314 (NKG2D; clone ON72) was purchased from Beckman Coulter. PE Mouse IgG1 (Clone 11711), PE Mouse Anti-Human CD155 (PVR; Clone 300907), Mouse IgG2b (Clone 133303), Mouse Anti-Human MICB (Clone 236511), Mouse IgG2A (Clone 20102), Mouse Anti-Human ULBP-1 (Clone 170818), Mouse Anti-Human ULBP-2/5/6 (Clone 165903), and Mouse Anti-Human ULBP-3 (Clone 166510) were purchased from R&D Systems. The cells were stained with their corresponding isotype control antibodies.

### NK cell-mediated cytotoxicity assay

K562, PANC-1, and MIA PaCa-2 cells were labeled with carboxyfluorescein succinimidyl ester (CFSE) at a final concentration of 5 μM at 37 °C for 10 min in a humidified incubator with 5% CO_2_. To evaluate the cytotoxic effects of gemcitabine and erlotinib, PANC-1 and MIA PaCa-2 cells were incubated with gemcitabine (vehicle distilled water) at final concentrations of 0, 10, 25, and 50 nM for 48 h, or erlotinib (vehicle DMSO) at final concentrations of 0, 0.1, and 10 µM for 24 h. After labeling, the cells were washed with complete medium. NK cells (effector cells) were co-cultured at 37 °C and 5% CO_2_ for 4 h with CFSE-labeled target cells at appropriate effector-to-target cell count ratios (E:T; 10:1, 5:1, 2.5:1). We then added 7-AAD Viability Staining Solution (BioLegend, San Diego, CA, USA) at 5 μL/test to label the DNA of dead cells. Dead cells were analyzed using flow cytometry.

### NK cell degranulation (CD107a) assay

NK cells were co-cultured with target cells (K562, PANC-1, and MIA PaCa-2 cells) at a ratio of 1:1 in a final volume of 200 μL at 37 °C and 5% CO_2_ for 4 h in the presence of FITC Mouse Anti-Human CD107a antibody (BD Biosciences) and monensin (GolgiStop; BD Biosciences) with brefeldin A (GolgiPlug; BD Biosciences). The cells were stained using PE Mouse Anti-Human CD3 and PE-Cy5 Mouse Anti-Human CD56 antibodies for 30 min. The cells were then washed and analyzed via flow cytometry.

### In vivo xenograft model analysis

Five-week-old female NOD/SCID (NOD.CB17-Prkdcscid/ARC) mice were purchased from Central Lab Animal Inc. (Seoul, South Korea). PANC-1 (2 × 10^6^ cells/mouse) and MIA PaCa-2 (2 × 10^6^ cells/mouse) human pancreatic cancer cells were subcutaneously inoculated into the left flank of each mouse. Gemcitabine (Gemzar injection, BORYUNG Pharmaceutical, Seoul, South Korea) and erlotinib (Tarceva Tab, Roche Korea, Seoul, South Korea) were administered simultaneously to the mice when the tumor grew to a volume of approximately 50–100 mm^3^. Gemcitabine was administered intraperitoneally once a week at a concentration of 20 mg/kg. Sulfobutyl ether β-CD (SBE-β-CD, cyclodextrin; MedChemExpress, Monmouth Junction, NJ, USA), a solvent for erlotinib, was diluted in water to a concentration of 6%. Erlotinib was administered daily via oral gavage at a dose of 50 mg/kg. Three days after gemcitabine administration, NK cells (1 × 10^7^ cells) were injected into the tail vein of the mice. The tumor volume (length × width^2^ × 0.5) was measured twice weekly. Gemcitabine and NK cell injections were administered three times at 1-week intervals. Tumor volume was without phase measured after 3 weeks of treatment.

### Immunohistochemistry

After 14 days of treatment, the tumors were isolated from the mice. Freshly collected tissues were snap-frozen using OCT Compound (Sakura, Tokyo, Japan) in liquid nitrogen. Tissue sections (6-μm-thick) were prepared, air-dried, and fixed for 10 min at 4 °C in methanol (Honeywell Research Chemicals, Seelze, Germany). Cryosections were blocked for 30 min with a biotin-blocking solution (Invitrogen; Thermo Fisher Scientific), washed in PBS, and incubated overnight at 4 °C with Biotin Anti-Human CD56 Antibody (Clone NCAM, Biolegend), Alexa Fluor 488-conjugated Streptavidin (Molecular Probes, Eugene, OR, USA), and PE-conjugated Ki-67 Monoclonal Antibody (Clone SolA15, eBioscience), as well as DAPI (Thermo Fisher Scientific). The sections were then washed with PBS and stained with DAPI. Immunohistochemistry images were captured using an upright microscope (ECLIPSE 80i, Nikon, Tokyo, Japan).

### RNA-seq

Using the method described above, freshly isolated resting NK cells and NK cells expanded for 21 d were harvested. RNA was extracted from two different healthy donors using TRIzol reagent (Invitrogen; Thermo Fisher Scientific) and RNA-seq was performed at Macrogen Inc. (Macrogen Inc. Seoul, South Korea). A sequencing library was established using the SureSelectXT RNA Direct Reagent Kit (Agilent Technologies, Santa Clara, CA, USA) and sequenced using a NovaSeq 6000 system (Illumina, San Diego, CA, USA). After mapping the preprocessed reads to the reference genome using HISAT2, aligned reads were generated. After conducting transcript assembly using StringTie, fragments per kilobase of transcript per million mapped reads (FPKM)/reads per kilobase of transcript per million mapped reads (RPKM), and transcript per kilobase expression (TPM) values were extracted. For genes identified as significantly differentially expressed genes (DEGs), functional classification of Gene Ontology, biological process (BP), molecular function (MF), and cellular component (CC) were analyzed using gProfiler (https://biit.cs.ut.ee/gprofiler/orth). The ExDEGA analysis tool (E-biogen Inc. Seoul, South Korea) was used for follow-up analysis of DEGs.

### Statistical analysis

Statistical analysis was performed using GraphPad Prism version 8 (Graphpad, San Diego, CA, USA). The normality of the distribution was tested using the Shapiro–Wilk test. For variables that were normally distributed, the paired *t*-test was used for paired analyses. For variables that were not normally distributed, the Wilcoxon matched-pairs test was used. Comparisons of more than two conditions were analyzed via one-way or two-way analysis of variance (ANOVA) with post-hoc Tukey’s correction. Data are presented as the mean ± standard deviation (SD). Differences were considered statistically significant at *P < 0.05, **P < 0.01, and ***P < 0.001.

## Results

### Gemcitabine has an anti-proliferation effect on pancreatic cancer cell lines in vitro and increases the anti-tumor activity of NK cells

**Figure 1:**
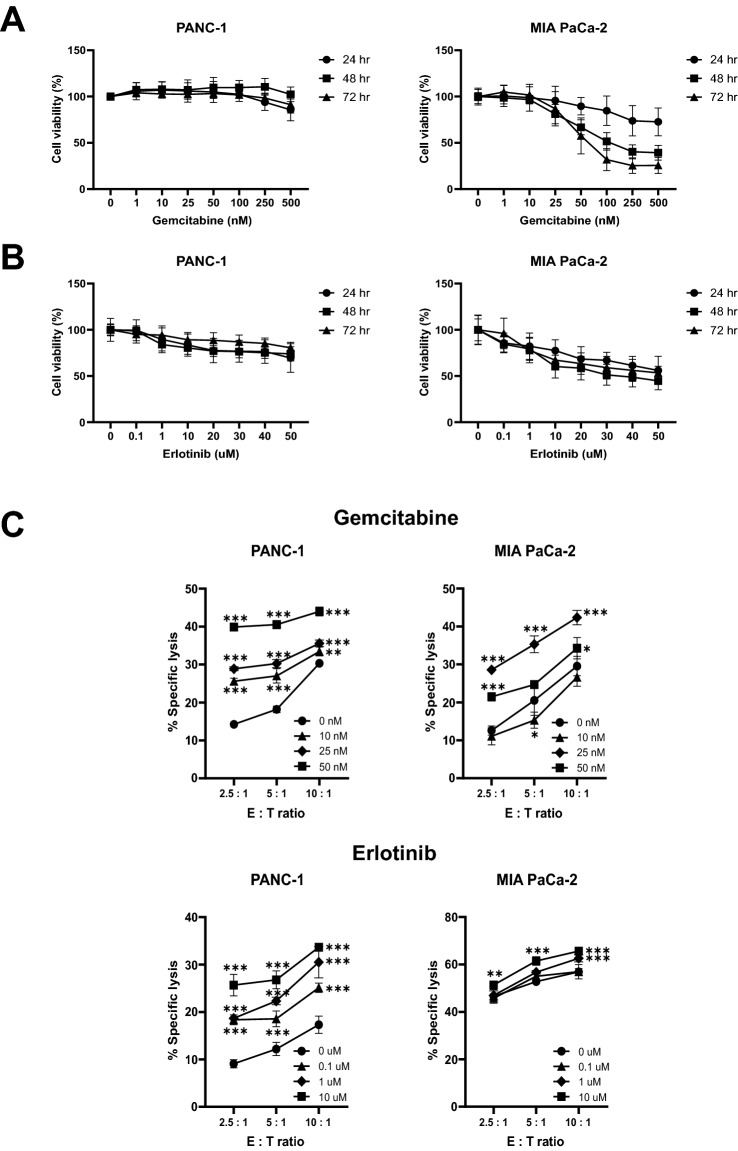
Effects of gemcitabine and erlotinib on the cytotoxicity of gemcitabine and NK cells. Gemcitabine (0–500 nM) **(A)** or erlotinib (0–50 μM) **(B)** were incubated with each pancreatic tumor cell line for 72 h, and cell viability was measured by adding WST reagent. Each pancreatic tumor cell line was treated with different concentrations of gemcitabine for 48 h or erlotinib for 24 h, then co-cultured with ex vivo-expanded NK cells, prior to NK cell cytotoxicity testing. Data are represented as means ± SD of at least three experiments. Statistical significance was determined via one-way ANOVA, relative to the control group, within each effector-to-target (E:T) cell ratio (**P < 0.01, ***P < 0.001) **(C)**.

To determine whether gemcitabine affects cell growth in pancreatic cancer cell lines in a concentration-dependent manner, the proliferation of these cell lines was analyzed using WST assay analysis after treatment with gemcitabine at various concentrations (0–500 nM) for 72 h. The proliferation of PANC-1 cells was not affected upon treatment with 500 nM gemcitabine. In contrast, MIA PaCa-2 cells showed sensitivity to gemcitabine (100 nM) and had a cell proliferation rate of 51.62% at 48 h (Fig. 1A). Therefore, to determine the concentration, we analyzed the results of the gemcitabine-sensitive MIA PaCa-2 cell line. We set the concentration of gemcitabine to not exceed 100 nM to avoid inhibiting cell growth. Additionally, we analyzed whether erlotinib concentrations inhibited cell growth. This revealed that the MIA PaCa-2 cell line was more sensitive to erlotinib than to PANC-1 cell line (Fig. 1B). We set the concentration of erlotinib to not exceed 20 µM, considering that erlotinib has an IC_50_ > 20 µM for the PANC-1 and MIA PaCa-2 cell lines^35^. We treated pancreatic cancer cell lines with gemcitabine for 48 h and co-cultured them with ex vivo-expanded NK cells. Gemcitabine increased the specific lysis of PANC-1 cells in a dose-dependent manner, whereas for the MIA PaCa-2 cells, specific lysis was highest at 25 µM gemcitabine.

We analyzed the specific lysis due to ex vivo-expanded NK cells after treatment of the pancreatic cancer cell lines with erlotinib for 24 h. Erlotinib increased the specific lysis of PANC-1 cells in a dose-dependent manner. For MIA PaCa-2 cells, the combination of high-dose erlotinib and a high E:T ratio achieved a higher specific lysis than any other combination (Fig. 1C). These results suggest that the combination of high-dose gemcitabine with a high E:T ratio was effective in the PANC-1 cell line, and the combination of high-dose erlotinib with a high E:T ratio was effective in the MIA PaCa-2 cell line.

### Treatment with gemcitabine and erlotinib alters NK cell sensitivity in pancreatic cancer cell lines

**Figure 2:**
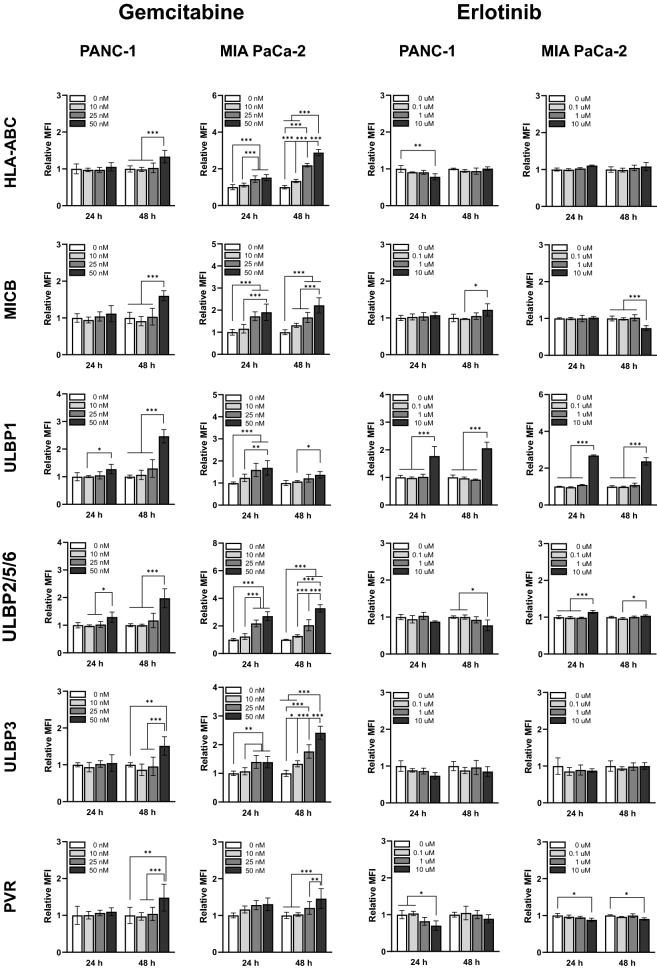
Effects of gemcitabine and erlotinib on NK cell recognition markers. After culturing the pancreatic tumor cell lines treated with gemcitabine (0, 10, 25, 50 nM) or erlotinib (0, 0.1, 1, or 10 µM) for 24 or 48 h, markers related to NK cell recognition were analyzed via flow cytometry. Relative median fluorescence intensity (MFI) was calculated as delta MFI (MFI test − MFI isotype control) divided by control MFI (zero concentration at 24 h). Data are represented as means ± SD of at least three experiments. Statistical significance was determined via two-way ANOVA (*P < 0.05, **P < 0.01, ***P < 0.001).

To evaluate whether chemotherapy induces the expression of inhibitory and activation receptors in NK cells, the cells were treated with gemcitabine (0, 10, 25, and 50 nM) or erlotinib (0, 0.1, 1, and 10 µM) and incubated for 24 or 48 h, and ligand expression was analyzed via flow cytometry. We observed an increase in the expression of HLA-ABC, a ligand of the inhibitory receptor KIR in NK cells, and in those of MICB, ULBP1, ULBP2/5/6, ULBP-3, and PVR, ligands of activation receptors, 48 h after treatment with 50 nM gemcitabine in all cell lines (Fig. 2). The MIA PaCa-2 cell line ligands we analyzed were sensitive to gemcitabine, with the highest increase in ULBP2/5/6 (3.2738-fold increase) achieved after 50 nM gemcitabine treatment for 48 h. Treatment with erlotinib tended to increase ULBP1 expression rather than that of other ligands in these cell lines. These results suggest that gemcitabine more directly induces ligand expression in MIA PaCa-2 cells, and that it is more efficient than erlotinib in inducing ligand expression in both cell lines.

### Ex vivo-stimulated and expanded NK cells have greater cytotoxicity against pancreatic cancer cells

**Figure 3:**
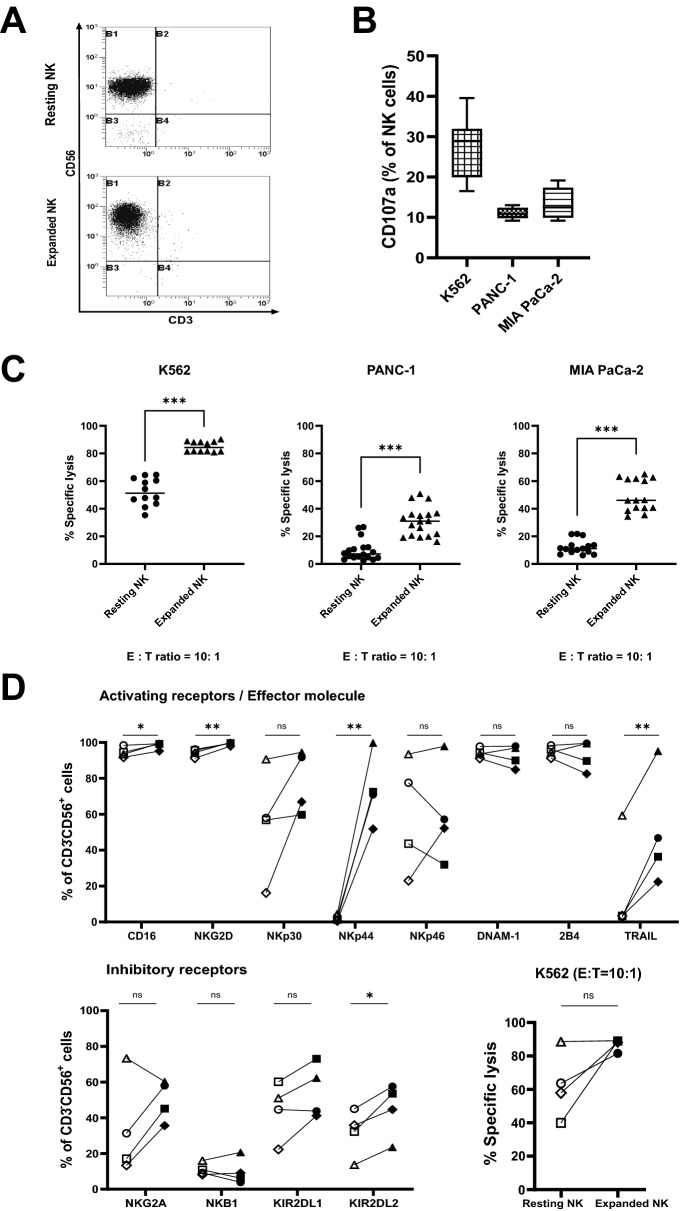
Characterization of resting and ex vivo-expanded NK cells. Representative flow cytometry dot plots of CD3^−^CD56^+^ resting and ex vivo-expanded NK cells. Resting NK cells: cells analyzed immediately after NK cell isolation **(A)**. CD107a expression in ex vivo-expanded NK cells co-cultured with K562, PANC-1, or MIA PaCa-2 cell lines **(B)**. Cytotoxicity of resting and ex vivo-expanded NK cells against their respective target cells. Data are represented as means ± SD of at least three experiments. Statistical significance was determined via paired *t*-test (***P < 0.001) **(C)**. Phenotypic analysis of resting and ex vivo-expanded NK cells. The analysis was performed using NK cells from four different healthy donors. Each symbol represents a donor and each connected symbol represents one sample. The symbol on the left of each marker indicates resting NK cells and the symbol on the right indicates expanded NK cells. Statistical significance was determined via paired *t*-test or Wilcoxon matched-pairs test, according to the normality of the distribution (Shapiro–Wilk test). (*P < 0.05, **P < 0.01; ns, not significant) **(D)**.

In a previous study, we demonstrated that the combination of irradiated PBMCs and anti-CD16 monoclonal antibodies synergistically increased the expansion of ex vivo-stimulated NK cells^36^. In this study, PBMCs were activated with CD3, IFN-γ, and IL-2, and then irradiated. Activated and irradiated PBMCs were then used as feeder cells to expand NK cells stimulated with anti-CD16 monoclonal antibodies. Following 21 days cell expansion, T-cell contamination of isolated NK cells and concentrated NK cells was less than 1% (Fig. 3A). The ex vivo-expanded NK cells were then co-cultured with pancreatic cancer cell lines, including the MHC class I-deficient myeloid leukemia cell line K562, and were evaluated via flow cytometry. Expanded NK cells exhibited greater anti-tumor cytotoxicity than resting NK cells (Fig. 3C). Relative to that of resting NK cells, expanded NK cell cytotoxicity was greater by 32.71 ± 3.04% in K562 cells, 21.06 ± 3.06% in PANC-1 cells, and 37.12 ± 3.24% in MIA PaCa-2 cells. Considering that NKG2D ligand expression is mostly higher in MIA PaCa-1 cells than in PANC-1 cells (Supplementary Fig. 1), these differences are to be expected.

We then analyzed expanded NK cell cytotoxicity toward their target cells via flow cytometry after co-culturing these cells for 4 h. Specifically, we examined the expression of CD107a, an indicator of NK cell activity found on the surface of NK cells (Fig. 3B). Soluble granules are degranulated on the NK cell surface; hence, degranulation by NK cells can be evaluated. Although there were differences between the donors, the expression of CD107a when co-cultured with the target cells was greater by 27.40 ± 7.38%, 11.07 ± 1.29%, and 13.37 ± 3.76% for K562, PANC-1, and MIA PaCa-2 cells, respectively.

Next, to investigate the receptors involved in the cytotoxicity of resting and expanded NK cells against their target cells, we analyzed the expression of activating and inhibitory receptors on the NK cell surface using flow cytometry. Figure 3D shows the surface expression of activating receptors (CD16, NKG2D, NKp30, NKp44, NKp46, DNAM-1, and 2B4) and inhibitory receptors (NKG2A, NKB1, KIR2DL1, and KIR2DL2) in both resting and expanded NK cells, as well as the surface expression of effector molecules (TRAIL). We analyzed the anti-tumor efficacy and changes in receptors expression for each healthy donor (n = 4) by separately for resting and expanded NK cells. Nkp44 and TRAIL expression was upregulated in cells from donors who exhibited high anti-tumor efficacy. Among the inhibitory receptors, KIR2DL2 exhibited the least upregulation. Together, these results show that NK cells expanded in vitro with CD3, IFN-γ, IL-2, and irradiated PBMCs as feeder cells had greater anti-tumor activity than resting NK cells. Further, they suggest that the upregulation of receptors such as NKG2D, NKp44, and TRAIL gives expanded NK cells their greater cytotoxic ability relative to that of resting NK cells.

### Combination of gemcitabine-based chemotherapy and in vitro-expanded NK cells exhibits potent anti-tumor effects against pancreatic cancer xenografts in NOD/SCID mice

**Figure 4:**
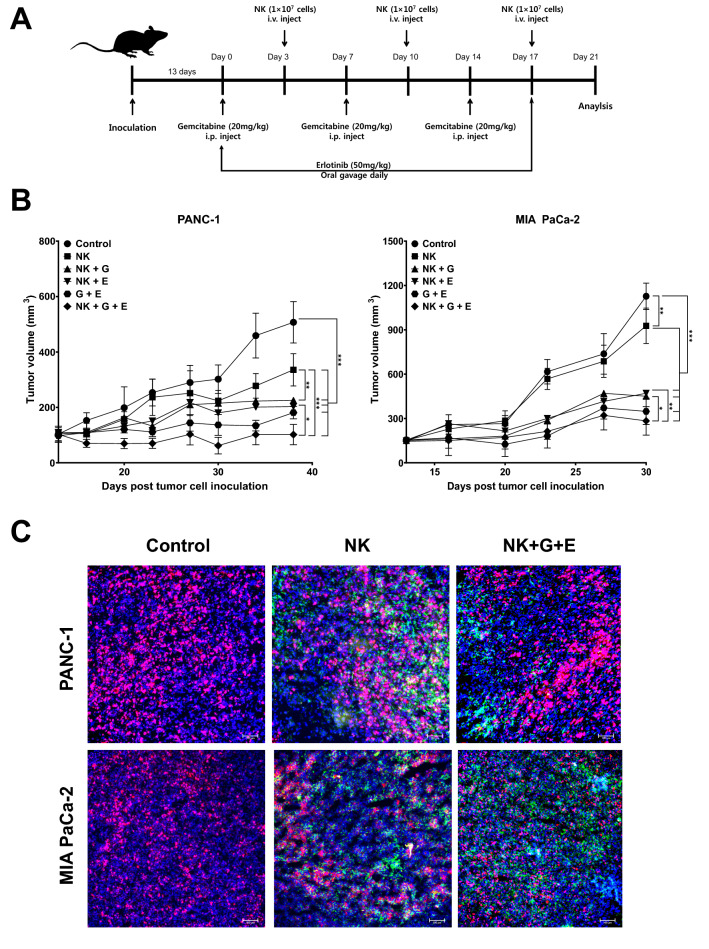
Effects of gemcitabine-based chemotherapy and ex vivo-expanded NK cells on pancreatic cancer xenografts. PANC-1 cells and MIA PaCa-2 cells were transplanted into the left flank of NOD/SCID mice. Schematic showing the animal study design. Ex vivo-expanded NK cells (1 × 10^7^) and gemcitabine (20 mg/kg) were administered once a week, and erlotinib (50 mg/kg) was administered daily **(A)**. Pancreatic cancer xenograft tumor volume. Results shown are average tumor volumes ± SD for five mice per group. Statistical significance was determined via one-way ANOVA (*P < 0.05, **P < 0.01, ***P < 0.001). *G* Gemcitabine, *E* Erlotinib. **(B)**. Representative images of intratumoral expression of various markers in ex vivo-expanded NK cells after intravenous injection. Fluorescence staining of CD56 (FITC), Ki-67 (PE), and DAPI (APC). Immunohistochemistry was analyzed in triplicate. Original magnification, × 100 **(C)**.

We evaluated the anti-tumor effects of a combination of gemcitabine, erlotinib, and expanded NK cells administered to pancreatic cancer xenograft NOD/SCID mice. PANC-1 and MIA PaCa-2 human pancreatic cancer cells were inoculated subcutaneously into the left flank of NOD/SCID mice. Mice were then intraperitoneally administered 20 mg/kg (G) gemcitabine once a week, and expanded NK cells were administered to the tail vein 3 days after gemcitabine administration. Erlotinib (E) was orally administered at 50 mg/kg daily. Tumor growth inhibition was observed for 3 weeks after treatment. The combined administration of expanded NK cells and chemotherapy inhibited tumor growth (Fig. 4B). In the PANC-1 xenograft model, tumor growth was significantly inhibited (*P* < 0.001) using NK cells alone, gemcitabine plus erlotinib, and NK cells plus chemotherapy, relative to the control group. Relative to using NK cells alone, using NK cells plus gemcitabine plus erlotinib (NK + G + E group) significantly inhibited tumor growth (*P* < 0.001). In the MIA PaCa-2 xenograft model, relative to the control group, NK cells alone, gemcitabine plus erlotinib, and NK cells plus chemotherapeutic agents significantly inhibited tumor growth. Relative to the control, tumor growth was significantly inhibited using NK cells alone (*P* < 0.01), while the other combinations exhibited more significant inhibition (*P* < 0.001). Remarkably, in these pancreatic cancer xenograft models, using NK cells plus gemcitabine plus erlotinib (NK + G + E group) achieved the greatest inhibition of pancreatic cancer tumor growth.

Next, we used immunohistochemistry to analyze the tumor tissues harvested 14 d after the last treatment administration, to further investigate the effects of NK cells on tumor growth inhibition. The human NK cell marker CD56 was expressed in the NK cell-administered mouse tumor tissue (Fig. 4C), suggesting that NK cells can infiltrate tumor tissues. Combining the results of NK cell invasion and tumor size, NK cells penetrated the tumor and exhibited tumor growth inhibitory activity even after NK cell administration was terminated.

### Gene expression signature of ex vivo-expanded NK cells using RNA sequencing

**Figure 5:**
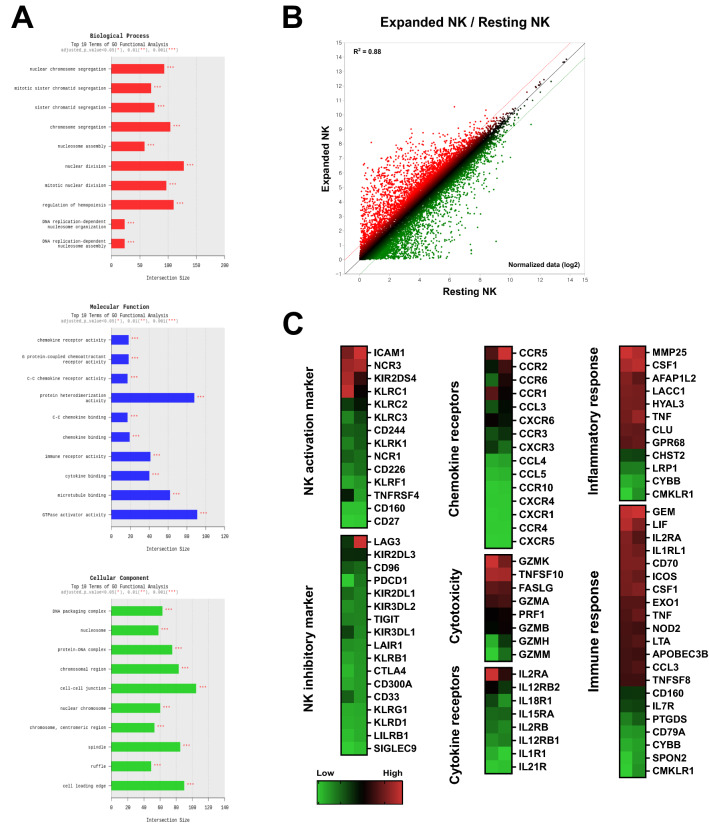
Gene expression signatures of resting and ex vivo-expanded NK cells, via RNA sequencing. Gene ontology analysis for biological processes, molecular functions, and cellular components **(A)**. Scatter plot of RNA sequencing data **(B)**. Heatmap illustrating gene clustering based on gene ontology analysis. Warmer colors (red) indicate higher gene expression and cooler colors (green) indicate lower gene expression. Each column represents the expression profile of one donor **(C)**.

We performed RNA sequencing to determine the gene expression profiles of NK cells expanded ex vivo relative to that of resting NK cells. Figure 5A shows the results of the gene ontology analysis of those DEGs satisfying at least one of the following: |fold change|≥ 2 and *P* < 0.05. Gene ontology biological process analysis showed that the upregulated DEGs were enriched in chromosome and nuclear chromosome segregation regulation, nuclear division regulation, and hematopoietic regulation. Molecular function analysis showed enrichment in GTPase activator activity and protein heterodimerization activity. Finally, cell–cell junctions were the most enriched in the cellular component analysis, followed by the chromosomal region and cell leading edge. Figure 5B illustrates the expression of transcripts significantly expressed in ex vivo-expanded NK cells relative to resting NK cells.

Next, we analyzed those genes whose expression was significantly different in ex vivo-expanded NK cells relative to resting NK cells using a more diverse gene ontology (Fig. 5C). Gene ontology analysis revealed elevated expression of NK activation markers (*ICAM1* and *NCR3*), NK inhibitory markers (*LAG3* and *KIR2DL3*), cytotoxicity markers (*GZMK, TNFSF10,* and *FASLG*), inflammatory response markers (*MMP25* and *CSF1*), chemokine receptors (*CCR5* and *CCR2*), cytokine receptors (*IL2RA*), and immune responses (*GEM, LIF, IL2RA,* and *IL1RL1*). These results suggest that our PBMC activation method for ex vivo expansion of NK cells achieves elevated expression of various NK cell activation receptors and cytotoxicity-related genes.

## Discussion

NK cells are divided into two subsets: CD56dim NK cells (ca. 90%), which express high levels of CD16 molecules and mainly exert a cytotoxic function, and CD56bright NK cells, which have low CD16 expression and predominantly exert immunomodulatory functions via cytokine secretion^37^. The activating receptor NKG2D binds to a family of ligands with structural homology to MHC class I, and the inhibitory receptor KIR recognizes HLA-A, HLA-B, and HLA-C proteins. NK cells regulate cytotoxic activity by transmitting cytotoxic signals via binding between their receptors and target-cell ligands^22^. Because of these characteristics, anticancer immune cell therapy based on NK cells does not require strong histocompatibility, unlike T-cell-based therapy^22^, and can complement the limitations of existing therapies, such as conventional surgical treatment, radiation therapy, and chemotherapy. This treatment has attracted attention owing to its advantages.

In this regard, the value of the safety and effectiveness of anticancer treatment using immune cell therapy has recently been recognized, and the number of related clinical papers is increasing. In a study published in 2014, the administration of a cytokine-induced killer cell (CIK) injection to patients with pancreatic cancer who failed first-line chemotherapy with gemcitabine showed a 25% response rate and a median survival rate of 26.6 weeks. This effect was comparable to that of chemotherapy^38^. In addition, in a study published in 2017, immunotherapy using NK cells cultured ex vivo with irradiated K562-mb15-41BBL cells as feeder cells, in patients who first received gemcitabine or FOLFIRINOX chemotherapy, resulted in an OS of 13.9 months^39^. Based on the results of previous studies, combining immune cell therapy and anticancer drugs can potentially overcome the limitations of primary anticancer drugs alone.

Gemcitabine regulates NKG2D ligand expression in pancreatic cancer cell lines. Gemcitabine-treated pancreatic cancer cells had increased expression of the NKG2D ligands MICA/B and ULBP2 on their surface^31^. In contrast, they exhibited reduced levels of soluble MICB and ULBP2 in the culture medium and of the metalloproteases ADAM-10 and ADAM-15 that control them^40,41^. In this study, gemcitabine upregulated the expression of MICB, ULBP1, ULBP2/5/6, ULBP3, and PVR in pancreatic cancer cell lines (Fig. 2). Erlotinib also upregulated ULBP1 (Fig. 2), and this upregulated ligand expression enhanced cytotoxicity when co-cultured with expanded NK cells (Fig. 1C). This is consistent with previous findings that low concentrations of gemcitabine increase the expression of MICA/B, the NKG2D ligand, in pancreatic cancer cell lines and elicit a potent apoptotic effect in NK cells^31^.

According to reports on the immunological activity of ex vi*vo*-expanded NK cells against pancreatic cancer, autologous NK cells derived from PDAC patients achieved increased specific lysis of autologous tumors (> 70%); allogenic NK cells from healthy donors exhibited lower but nonetheless substantial specific lysis (> 60%). Further, a significant reduction in tumor size has been observed in in vivo models^42^. After administering NK cells to an orthotopic transplantation mouse pancreatic cancer model, pancreatic cancer treatment reduced tumor volume at least fourfold relative to gemcitabine treatment alone^43^. In our study, the NK cell plus gemcitabine plus erlotinib treatment group (NK + G + E group) had 4.69-fold fewer tumors than the PANC-1 xenograft control group and 3.97-fold fewer than the MIA PaCa-2 xenograft control group (Fig. 4B). Moreover, NK cells successfully infiltrated desmoplastic pancreatic tumors and exhibited anticancer activity even after treatment termination (Fig. 4C). NK cells could infiltrate the tumor microenvironment, and it is critical to identify interactions between tumor cell ligands and NK cell receptors. An immunohistological analysis of NK cell infiltration in a human ovarian cancer xenograft mouse model used NKG2D, rather than CD56, as the NK cell tumor-infiltration marker^44^. In addition, although it used a human renal cell carcinoma sample, immunohistological analysis of NK cell infiltration of this tissue revealed CD56 staining at the same location as the NK cell-activating receptors NKp46 and NKp30^45^. Based on these reports, CD56 staining, which was an important marker for NK cell infiltration in our study, could be associated with the presence of other promising NK cell receptors. As tumor-infiltrating NK cells are an indicator of the prevention of recurrence and improved survival, NK cell infiltration plays an important role in the anti-tumor immune response.

NK cells from patients with malignant pancreatic cancer are reported to have lower cancer cell-killing ability than those from patients with benign pancreatic tumors^46^. Further, progressive impairment of NK cell function in patients with pancreatic cancer is associated with pancreatic cancer stage. A defect in CD107a was observed in patients with pancreatic cancer, but was absent in normal subjects and patients with benign pancreatic tumors^46^. Regarding the representative gene expression patterns, NK cells expanded ex vivo using the method described in this study characteristically expressed high levels of CD54 (*ICAM1*, 9.53-fold increase), NKp30 (*NCR3*, 9.56-fold increase) and CCR5 (*CCR5*, 9.23 × 10^11^-fold increase) demonstrating their improved cytotoxic activity (Fig. 5C). Although it was not observed in the gene expression patterns, a surprising upregulation of NKp44 was observed (Fig. 3D). A prior RNA sequencing study of ex vivo-expanded NK cells after cryopreservation reported the upregulation of NKp44, CD40L, and CCR5 expression^47^. Further, RNA sequencing of NK cells ex vivo-expanded with IL-2 revealed upregulation of genes related to chemokine receptors and cytotoxicity markers^48^. These results suggest that ex vivo expansion of NK cells can improve NK cell function.

Preclinical development of NK cell-based therapy has been pioneered in the direction of expanding NK cell capacity for therapy and elucidating how NK cells are activated. As methods to activate and expand in vitro allogeneic NK cells for adoptive transfer have improved, protocols for the preparation of clinical-grade NK cells have been established^49^. An optimized protocol has been presented for the enrichment of CD3^−^CD56^+^ cells along with high levels of purification to limit T-cell contamination, prior to the proliferation of donor-derived NK cells^49^. Our results revealed < 1% T-cell contamination, with > 95% NK cell purity after expansion (Fig. 3A). A number of Phase I and Phase I/II clinical trials are ongoing for NK cell immunotherapy to treat solid cancers, including pancreatic cancer^50^. Increasing numbers of clinical trials are testing the use of autologous NK cells, allogenic NK cells, and CAR–NK therapy, alone or in combination with other treatments.

Our study relied on NOD/SCID mice. Therefore, further validation of our findings is required in animal models that are highly relevant to humans or human subjects. Although our study provides valuable insights into the interactions between NK cells and tumor cells, further studies are needed to fully evaluate the translational and clinical relevance of our findings.

Our findings demonstrate that irradiation of stimulated autologous PBMCs (the feeder cells) induced NK cell proliferation and activation. The NK cells exhibit differences in cell surface markers and cytotoxic ability depending on the culture method^51–53^. Here, we have suggested an advanced NK cell culture method to achieve better cytotoxic ability relative to that of resting NK cells. Ex vivo-expanded NK cells combined with gemcitabine and erlotinib significantly inhibited tumor growth in a pancreatic tumor xenograft model, exhibiting high in vitro toxicity. Although prior studies have reported that the combined use of NK cells and gemcitabine is useful for increasing cytotoxicity in pancreatic cancer^29,30,54,55^, these studies did not include erlotinib in combination with gemcitabine. Our study, in contrast, reveals that the combination of gemcitabine and erlotinib enhanced NK cell cytotoxicity.

Our findings reveal that the concomitant use of gemcitabine upregulated the ligands of NK cell activation receptors, thus enhancing NK cell cytotoxicity. The gene expression patterns of expanded NK cells prepared using our method had enhanced expression of activating receptors and cytotoxicity relative to that of resting NK cells. Further, we showed that efficient infiltration by NK cells sustained their anti-tumor immune response. These findings reveal that activated NK cells can efficiently suppress pancreatic tumors in this mouse model when used in combination with gemcitabine-based therapy.

## Supplementary Information

Supplementary Figure 1.

## Data Availability

RNA-seq data have been deposited in the NCBI GEO database (accession number: GSE222740, https://www.ncbi.nlm.nih.gov/geo/query/acc.cgi?acc=GSE222740, token number: wjahaocchnqtbiv).
