# Supplementary material for: Combinatorial immunotherapy with gemcitabine and ex vivo-expanded NK cells induces anti-tumor effects in pancreatic cancer
Source: Sci Rep. 2023 May 11;13:7656. doi: 10.1038/s41598-023-34827-z (PMC10175562; doi:10.1038/s41598-023-34827-z)
Supplement: Supplementary file 1 — Supplementary Figure 1. [file 41598_2023_34827_MOESM1_ESM.docx]

**Supplementary information**


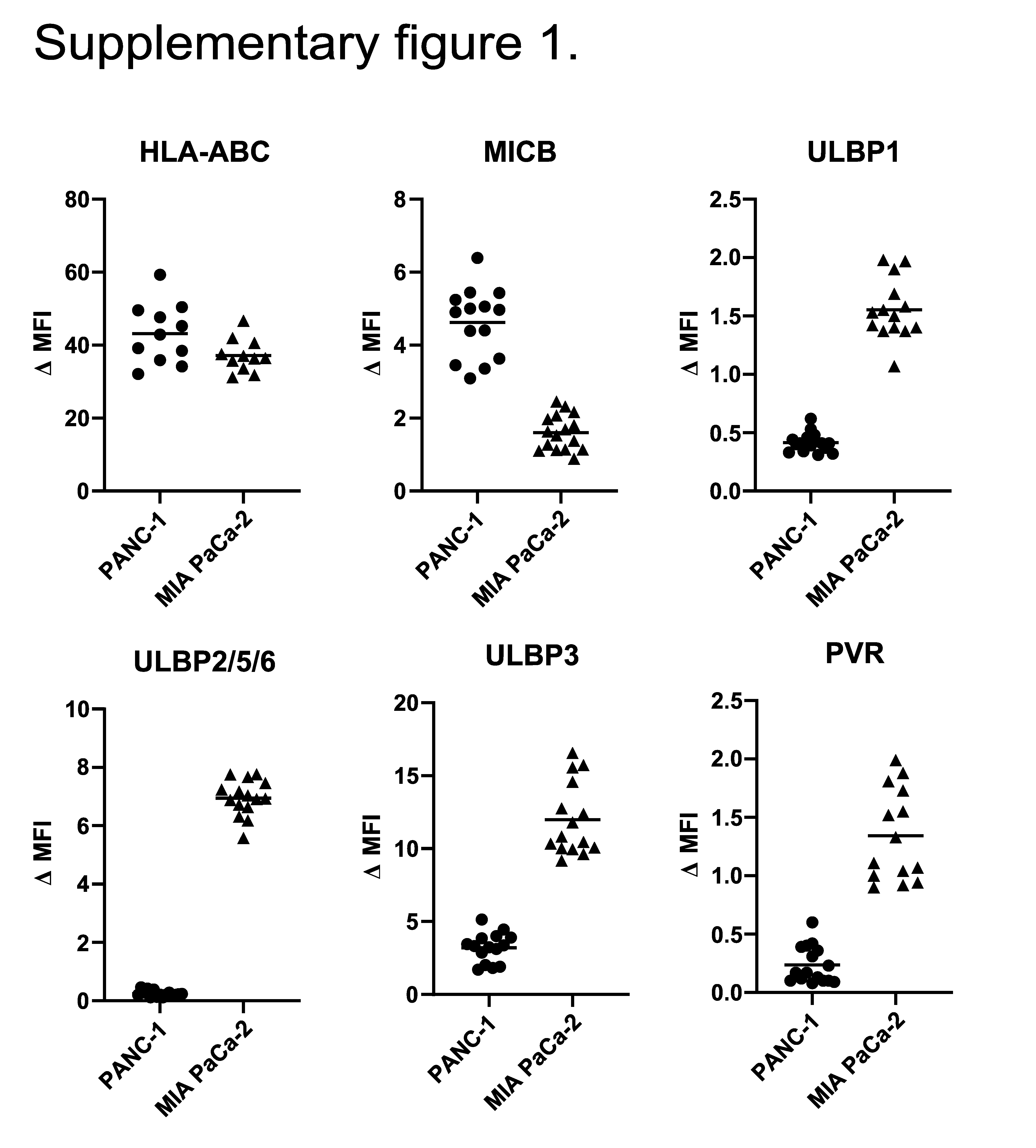


**Supplementary figure legend 1.** The NKG2D ligand of PANC-1 and MIA PaCa-2 pancreatic cancer cells. After culturing pancreatic tumor cell lines for 24 hours, NKG2D ligands were analyzed by flow cytometry. Delta Median fluorescence intensity (ΔMFI) was calculated as “MFI test – MFI isotype control”. Each symbol represents one sample. The analysis was performed at least triplicate experiments.
